# Hidden carriers: multidrug-resistant bacteria in hedgehogs from a wildlife rescue centre

**DOI:** 10.3389/fvets.2026.1754930

**Published:** 2026-01-22

**Authors:** Martina Masarikova, Aneta Papouskova, Darina Cejkova, Minoo Partovi Nasr, Iva Sukkar, Alois Cizek

**Affiliations:** 1 Faculty of Veterinary Medicine, Institute of Infectious Diseases and Microbiology, University of Veterinary Sciences Brno, Brno, Czechia; 2 Faculty of Electrical Engineering and Communication, Department of Biomedical Engineering, Brno University of Technology, Brno, Czechia; 3 Central European Institute of Technology, University of Veterinary Sciences Brno, Brno, Czechia; 4 Faculty of Medicine in Pilsen, Department of Microbiology, Charles University, Pilsen, Czechia

**Keywords:** antimicrobial resistance, *Escherichia coli*, hedgehog, one health, whole-genome sequencing

## Abstract

**Introduction:**

Antimicrobial resistance (AMR) represents a growing One Health challenge at the human–animal–environment interface. Wildlife rescue centres may represent potential, underrecognized settings where resistant bacteria could emerge and disseminate due to close human–animal contact and antimicrobial use. We investigated AMR profiles and genomic features of *Escherichia coli* isolated from European and northern white-breasted hedgehogs (*Erinaceus europaeus*, *Erinaceus roumanicus*) admitted to a Czech wildlife rescue facility.

**Materials and methods:**

Faeces from 23 hedgehogs were collected during routine pen cleaning. *E. coli* isolates were obtained on MacConkey agar (MCA) and MCA with cefotaxime and confirmed by MALDI-TOF MS. Antimicrobial susceptibility to 13 antibiotics was assessed using the disc diffusion test. A subset of 26 isolates representing diverse resistance profiles was further characterised by whole-genome sequencing (WGS). Genomic analyses focused on sequence types, phylogroups, resistance genes, plasmid replicons, and virulence-associated genes.

**Results and discussion:**

More than half of the isolates (37/69; 54%) were multidrug-resistant, with resistance most frequently observed to ampicillin and nalidixic acid. No cefotaxime-resistant isolates or genes encoding extended-spectrum beta-lactamases or carbapenemases were detected. Whole-genome sequencing revealed substantial genetic diversity, including several sequence types that are commonly associated with human and animal infections, such as ST457, ST162, and ST624. Isolates carried a wide range of resistance determinants, including *bla*_TEM-1_ and *qnrB2* genes, plasmid replicons, and virulence-associated genes, with phylogroup F showing the highest virulence gene content. Despite the modest sample size, our findings indicate that hedgehogs in rehabilitation settings can act as reservoirs of multidrug-resistant *E. coli* with diverse genomic backgrounds, contributing to the environmental dissemination of AMR. The presence of sequence types and resistance genes commonly associated with human and veterinary infections further supports the relevance of rehabilitated wildlife to the broader epidemiology of AMR. Enhancing biosafety practices and antimicrobial stewardship in wildlife rescue operations is therefore essential to mitigate zoonotic risks within a One Health framework.

## 1 Introduction

Antimicrobial resistance (AMR) is one of the most serious challenges facing human and veterinary medicine today (1, 2). Its spread among humans, animals, and the environment poses a significant threat to the effectiveness of treatment and requires a multidisciplinary approach based on the One Health principle (3–5).

In recent decades, the contact between wild animals and the human environment has increased significantly, and hedgehogs (*Erinaceus europaeus, Erinaceus roumanicus*) are no exception (6). These synanthropic insectivores are commonly found in suburban and urban areas, where food sources and shelter are more available (7). At the same time, injuries caused by road traffic, lawn mowers, or attacks by domestic pets are frequently documented, leading to their regular admission to wildlife rescue centres and veterinary clinics (8). In these facilities, animals are often treated with systemic antibiotics commonly used in veterinary practice, including beta-lactams and fluoroquinolones. Such therapeutic intervention can impose selective pressure on the intestinal microbiota of hedgehogs, including naturally occurring enterobacteria, promoting the proliferation of resistant strains (9).

After recovery, hedgehogs are usually released back into the wild, where contact with soil, water, and other animals facilitates the dissemination of faecal bacteria. This environment might enable the horizontal transfer of resistant genes through mobile genetic elements such as plasmids and integrative and conjugative elements, which significantly contribute to the spread of antimicrobial resistance among microorganisms (10–12). This phenomenon is of particular concern in the context of transmissible mobile genetic elements carrying genes for extended-spectrum beta-lactamases (ESBL), carbapenemases, or plasmid-mediated quinolone resistance (PMQR) genes (13).

The diversity of *E. coli* strains colonizing wildlife hedgehogs remains largely unknown; we lack data on the occurrence of “risk” lineages, which are characterized by an ability to spread effectively, gain antimicrobial resistance, occasionally cause extraintestinal disease, and swich host species. We hypothesize that under specific conditions of rescue stations (the concentration of susceptible animals and the selective antibiotic pressure), the healthy diversity of colonizing microflora may decrease, and such lineages may have an advantage. The aim of this study was therefore to perform a comprehensive genomic characterization of *Escherichia coli* isolated from hedgehogs housed at a Czech rescue centre using whole-genome sequencing (WGS), focusing on antimicrobial resistance and virulence patterns.

The analysis includes the identification of resistance genes, mobile genetic elements, and the profiling of virulence-associated genes (VAGs). Based on multilocus sequence typing (MLST) and phylogenetic analysis, it is evaluated whether sequence types linked to human and livestock infections are present in these isolates. Overall, this study contributes to understanding the genetic plasticity of *E. coli* in wild hedgehogs and evaluates whether these synanthropic animals can serve as reservoirs of potentially high-risk clones capable of circulating between animals, humans, and the environment.

## 2 Materials and methods

### 2.1 Sample collection

In January 2020, a single faecal sampling was conducted on hedgehogs (*Erinaceus europaeus, E. roumanicus*) housed at a wildlife rescue station in Brno (South Moravia, Czech Republic) specialising in hedgehog rehabilitation. The animals originated from the city of Brno and its immediate surroundings, representing a geographically limited and relatively homogeneous urban–suburban area within a maximum distance of 30 km from the rescue facility. The sampled hedgehogs represented a range of ages and body weights; adult individuals admitted due to injury weighed between 700 and 1,100 g, while juveniles admitted because of insufficient body mass to survive the winter weighed less than 500 g. Upon arrival, each hedgehog underwent a veterinary examination, after which an appropriate treatment plan was initiated. Depending on the clinical condition, animals received conservative or surgical care, commonly accompanied by systemic antimicrobial therapy (see Supplementary Table 2). After successful recovery, hedgehogs were released back into the wild; individuals with permanent impairments that prevented survival remained indefinitely at the facility.

Faecal samples were collected by the rescue staff during routine pen cleaning for parasitological examination, without any manipulation of the housed animals, thereby avoiding stress. Fresh faecal material not needed for parasitological testing was subsequently provided to the Institute of Infectious Diseases and Microbiology (University of Veterinary Sciences, Brno, Czech Republic) for further laboratory processing. In total, 23 faecal samples were obtained, each representing a single hedgehog.

### 2.2 Isolation of *Escherichia coli*

For the *E. coli* isolation, collected faecal samples were thoroughly mixed and homogenised, and 1 gram of each sample was transferred into 10 mL of buffered peptone water (Oxoid, UK) and incubated for 24 h under aerobic conditions. After incubation, 10 μL of enrichment culture was streaked parallel onto (1) McConkey agar (MCA) for isolation of *E. coli* regardless of their resistance phenotype and (2) McConkey agar supplemented with 2 mg. L^- 1^ of cefotaxime (MCA_cef_) (Sigma-Aldrich, Germany) to detect cefotaxime-resistant *E. coli*. All inoculated plates were incubated under aerobic conditions at 37 °C for 48 h. Lactose-positive colonies with distinct morphologies (up to three from MCA and up to three from MCA_cef_) were subcultured onto Columbia blood agar (Oxoid, UK) and incubated under the same conditions. Only lactose-positive colonies were chosen because *E. coli* is typically lactose-fermented, forming pink colonies on MacConkey agar, which allows reliable differentiation from non-target enterobacteria. Identification of isolates was performed using MALDI-TOF MS on a Microflex LT device equipped with MALDI Biotyper software version 3.1 (Bruker Daltonics, Germany). Confirmed *E. coli* isolates were preserved at −80 °C in 0.5 mL of cryoprotective medium containing bacteriological peptone and glycerol.

### 2.3 Determination of antimicrobial resistance phenotypes

The susceptibility of *E. coli* isolates to 13 antibiotics was assessed using the disc diffusion test (DDT). The methodological procedure, interpretation of results, and use of the *E. coli* ATCC® 25,922 as a control strain were in accordance with the Clinical and Laboratory Standards Institute (CLSI) guidelines (14). The antibiotics tested (Oxoid, UK) included beta-lactams (amoxicillin–clavulanic acid 30 μg, ampicillin 10 μg, cephalothin 30 μg, cefoxitin 30 μg, ceftazidime 10 μg), aminoglycosides (gentamicin 10 μg, streptomycin 10 μg), fluoroquinolones/quinolones (ciprofloxacin 5 μg, nalidixic acid 30 μg), sulfonamides/trimethoprim (sulfamethoxazole-trimethoprim 25 μg, sulfonamide compound 300 μg), and other classes, including chloramphenicol (30 μg) and tetracycline (30 μg).

### 2.4 Whole-genome sequencing, assembly, and data analysis

Genomic DNA was isolated using the NucleoSpin Tissue Kit (Macherey-Nagel, Germany) and sequenced with a high-throughput short-read approach. Libraries were prepared using the Nextera XT DNA Library Preparation Kit (Illumina, USA) and sequenced on the NovaSeq 6,000 platform (Illumina) at the UTS Core Sequencing Facility, Ithree Institute, Australia. Raw paired-end Illumina reads were quality-processed with Trimmomatic v0.39 (15) to remove adapter sequences and regions of low quality (Q ≤ 20). *De novo* genome assembly was performed using Shovill v1.0.9 (https://github.com/tseemann/shovill).

The sequence type of isolates was determined using MLST 2.0 (16) with the *E. coli* database (https://mlst.warwick.ac.uk/mlst/), serotype using SeroTypeFinder 2.0 (17), and phylogroup using ClermonTyping (18). Genomic sequences were analysed via ABRicate v1.0.1 (https://github.com/tseemann/abricate) to determine antibiotic resistance genes, plasmid replicons, and virulence-associated genes using ResFinder released 2025-05-15 (19), PlasmidFinder v2.1 released 2025-04-09 (20), and VirulenceFinder 2.0 (21) databases, respectively. The threshold criteria were set to ≥90% identity over 90% coverage of reference sequences.

Phylogenetic relatedness among genomes was reconstructed using Prokka-annotated v1.14.6 (22) sequences, the core-genome multi-FASTA alignment was created with PIRATE v1.0.5 (23), and a maximum-likelihood tree was built using RAxML v8.2.13 (24) with the general time-reversible (GTR) model supported by 500 bootstraps. The matrices of single-nucleotide polymorphism (SNP) distances were obtained from the core-genome alignment using snp-dists (https://github.com/tseemann/snp-dists). The resulting tree was visualized in iTOL v5 (25).

The data are now available under BioProject ID PRJNA1368502 (https://www.ncbi.nlm.nih.gov/bioproject/1368502).

## 3 Results

### 3.1 Antimicrobial resistance phenotypes of *Escherichia coli* isolates from hedgehogs

From 23 faecal samples collected from hedgehogs, a total of 69 *E. coli* isolates were recovered on non-selective MacConkey agar, with three colonies representing each animal. Notably, we did not isolate any cefotaxime-resistant *E. coli* by culturing faeces on MCA_cef_.

Resistance to at least one antibiotic was observed in at least one of the three *E. coli* isolates from 22 of the 23 hedgehogs (see Supplementary Table 1). In hedgehog no. 190, two isolates were susceptible to all tested antibiotics, while the third was resistant only to cephalothin. In hedgehog no. 192, one isolate exhibited resistance to seven antibiotics. In six individuals, one of the three isolates was fully susceptible. Multidrug resistance (MDR)—defined as resistance to at least three different antibiotic classes—was detected in at least one isolate from 14 hedgehogs (14/23; 61%), accounting for 37 of the 69 isolates (54%).

Overall, the most frequent resistances were to ampicillin (50/69; 72%) and nalidixic acid (37/69; 54%), followed by ciprofloxacin, streptomycin, sulfonamide compound, and sulfamethoxazole-trimethoprim (33/69; 48%). Resistance to other antibiotics was as follows: tetracycline (30/69; 43%), cephalothin (25/69; 36%), amoxicillin-clavulanic acid (9/69; 13%), chloramphenicol (7/69; 10%), gentamicin (5/69; 7%), and cefoxitin (2/69; 3%). All isolates were susceptible to the third-generation cephalosporin ceftazidime. A detailed overview of resistance phenotypes is provided in Supplementary Table S1.

### 3.2 WGS-based characterization of *Escherichia coli* isolates from hedgehog: sequence types, phylogroups, and serotypes

Twenty-six isolates from 21 hedgehogs were selected for whole-genome sequencing (WGS) based on differences in their antimicrobial resistance profiles. Eight different sequence types (STs) were identified. ST457 (7/26; 27%) and ST162 (5/26; 19%) were the most prevalent, followed by ST624 (4/26; 15%), ST212 (3/26; 12%), ST224 (3/26;12%), ST973 (2/26; 8%), and ST58 and ST2448 (1/26 each; 4%).

**Figure 1:**
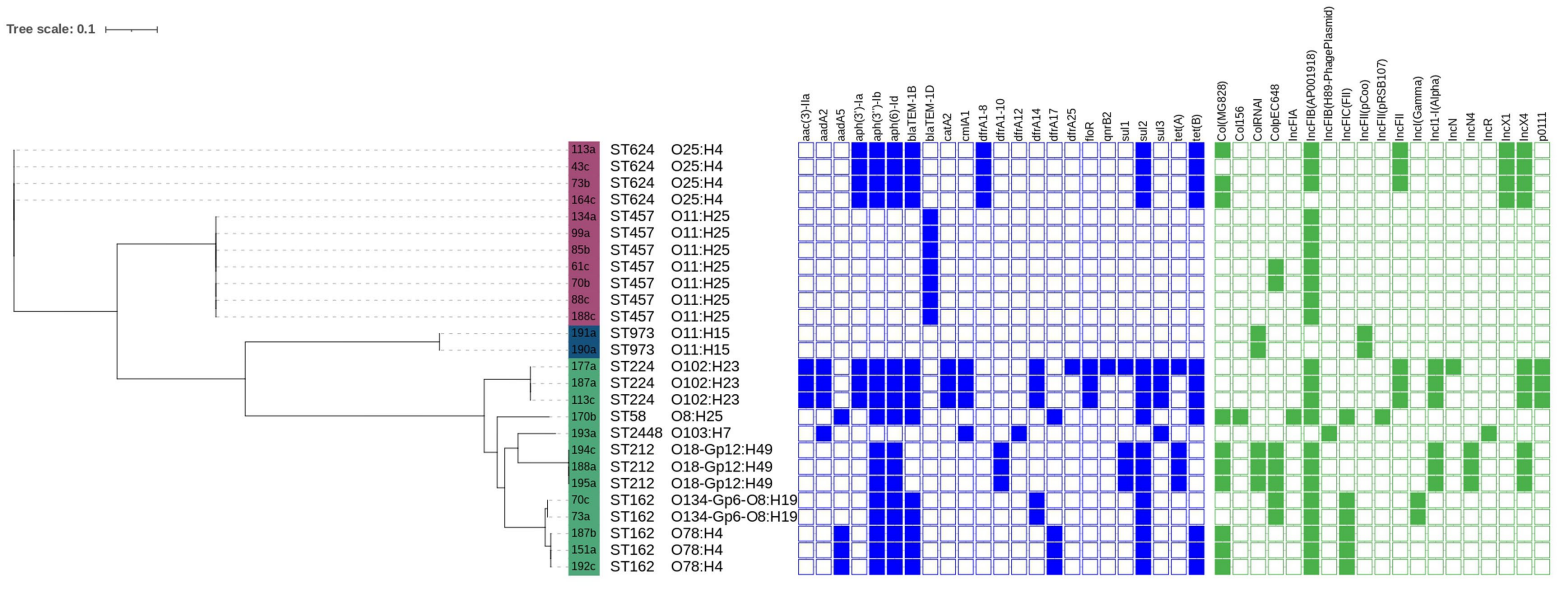
Phylogenetic analysis and genomic characterization of 26 *Escherichia coli* isolated from hedgehogs (*Erinaceus europaeus, Erinaceus roumanicus*) admitted to a Czech wildlife rescue centre. Phylogenetic groups are color-coded: F (purple), D (blue), and B1 (green). Antimicrobial resistance genes are indicated in blue, and plasmid replicon types are shown in green.

Phylogenetic analysis assigned the isolates to three groups: B1 (13/26; 50%), D (2/26; 8%), and F (11/26; 42%). Nine serotypes were identified, with O11: H25 (7/26; 27%) and O25: H4 (4/26; 15%) being the most common. Other serotypes were represented by 1–3 isolates of each (see Figure 1 for details).

### 3.3 Resistance genes and plasmid replicons

Genomic analysis confirmed the presence of a broad range of antimicrobial resistance genes, largely corresponding to the phenotypic resistance profiles determined by disc diffusion testing. Resistance determinants associated with multiple antimicrobial classes were identified, including genes conferring resistance to beta-lactams (*bla*_TEM-1B_, *bla*_TEM-1D_), aminoglycosides [*aac(3)-IIa*, *aadA2*, *aadA5*, *aph(3′)-Ia*, *aph(3″)-Ib*, *aph(6)-Id*], phenicols (*cmlA1*, *catA2*, *floR*), sulfonamides (*sul1*, *sul2*, *sul3*), trimethoprim (*dfrA1, dfrA12, dfrA14, dfrA17, dfrA25*), tetracyclines [*tet(A)*, *tet(B)*], and quinolones (*qnrB2*). No genes encoding ESBL were detected.

Beta-lactam resistance was predominantly mediated by *bla*_TEM_ variants, which were detected in the majority of sequenced isolates and were consistent with the high prevalence of ampicillin resistance observed phenotypically. Genes encoding resistance to aminoglycosides, sulfonamides, trimethoprim, and tetracyclines were frequently detected and often co-occurred within the same isolates, reflecting the multidrug-resistant phenotypes identified in several sequence types. Phenicol resistance genes were detected less frequently but were present in isolates exhibiting broader resistance profiles. Plasmid-mediated quinolone resistance was rare, with *qnrB2* identified in a single isolate only.

A diverse set of plasmid replicon types was identified across the sequenced isolates, with each genome carrying between one and seven replicons. IncF plasmids were the most prevalent and occurred across multiple phylogroups and sequence types, frequently in combination with other incompatibility groups. Additional plasmid types included IncI, IncN, IncX, and Col-like plasmids, highlighting the extensive plasmid diversity present within the *E. coli* population colonising hedgehogs in the rescue centre.

The distribution of resistance genes and plasmid replicons varied among sequence types and phylogenetic groups, suggesting multiple genetic backgrounds contributing to antimicrobial resistance within the studied population. Detailed isolate-level information on resistance genes and plasmid content is provided in Supplementary Table S2, while Figure 1 offers an integrated overview of phylogenetic relationships, resistance determinants, and plasmid replicons across the sequenced isolates.

### 3.4 Virulence-associated genes

All 26 sequenced isolates carried diverse VAGs (Supplementary Table S2). The lowest VAG content was observed in a B1-ST2448-O103: H7 isolate (two genes) and two D-ST973-O11: H15 isolates (four genes). Isolates from phylogroup F displayed the highest virulence gene content, with ST457-O11: H25 harboring seven genes and ST624-O25: H4 harboring nine. The most prevalent VAGs were *gad* (100%), *lpfA* (92%), *mchF* and *iss* (88%), and *iroN* (85%), followed by air and *eilA* (50%), *cma* (23%), *astA, mcmA*, and *ireA* (15%), *iha, celb, mchB*, and *mchC* (4%), and *tsh* (1%).

### 3.5 Phylogenetic structure of *E. coli* isolates

Phylogenetic reconstruction showed that the isolates were segregated into three primary lineages corresponding to phylogroups B1, D, and F. Phylogroup D formed a compact cluster with short branch lengths, indicating close relatedness. Phylogroup F was more dispersed, reflecting greater diversity within this lineage. A large assemblage dominated by ST624 and ST457 represented a polyphyletic group, suggesting multiple independent evolutionary origins. In contrast, phylogroup B1 exhibited deeper branching and higher heterogeneity, consistent with a more divergent lineage. Overall, phylogroups D and F were more closely related to each other than to the more distantly branching B1 group (Figure 1).

## 4 Discussion

In this study, we aimed to investigate the level of antimicrobial resistance of *E. coli* isolates colonizing hedgehogs in a small rescue station. From a One Health perspective, wildlife rescue centres represent critical interfaces where close contact between animals, humans, and the environment may facilitate bidirectional bacterial exchange. Hedgehogs may introduce antimicrobial-resistant *E. coli* into the facility, but they may also acquire new strains during their stay as a consequence of environmental exposure, antimicrobial treatment, or indirect contact with other animals and staff. Importantly, rehabilitated individuals are subsequently released back into the wild, which may contribute to the dissemination of resistant bacteria into the environment, highlighting wildlife rescue centres as potentially relevant but largely overlooked nodes in the ecology of antimicrobial resistance.

Although direct contact between individuals is minimized by housing each hedgehog in a separate enclosure and using dedicated husbandry equipment, indirect transmission of bacterial colonizers cannot be fully ruled out. The absence of environmental sampling within the rescue facility is acknowledged as a limitation of this study, as it precluded assessment of the contribution of environmental contamination to intestinal colonisation in hospitalised hedgehogs. Environmental samples could not be collected because access to the facility environment was not granted, and the study was therefore conducted exclusively using residual faecal material obtained after routine parasitological examination.

*E. coli* is a traditional indicator organism for monitoring antimicrobial resistance (26), as it is a widespread and versatile bacterium that colonizes the intestines of nearly all vertebrates and, second, the environment. Its remarkable adaptability is largely attributed to horizontal gene transfer involving mobile genetic elements (MGEs). Consequently, plasmid- or integron-associated resistance genes such as those encoding ESBL and AmpC beta-lactamases are considered to be of high epidemiologic significance. European hedgehogs, as free-ranging omnivores that feed on earthworms, have been proposed as sentinels for environmental antimicrobial resistance to pollution in their habitats (13).

Owing to the health conditions requiring their admission, the animals are highly susceptible to opportunistic infections and routinely undergo intensive treatment, including surgery and antimicrobial (AM) therapy. All sampled individuals received at least two AMs. Of the 21 animals (i.e., hedgehogs from which *E. coli* originated for WGS), 17 were treated with metronidazole + spiramycin (MTZ, SPIR), while amoxicillin-clavulanic acid (AMC), marbofloxacin (MAR), and sulfamethoxazole-trimethoprim (SXT) were used in eight animals in various combinations. Four animals received four different AMs, and one animal received all AMs listed. These drugs were administered sequentially for different clinical indications, not simultaneously (see Supplementary Table S2).

Despite this intense AM treatment, no ESBL-producing strains were detected. This may simply reflect the low number of sampled animals. Nevertheless, all isolates except three carried the *bla*_TEM-1_ gene and were resistant to ampicillin, and 17 were multidrug-resistant (MDR). Besides SXT, resistance to aminoglycosides and tetracycline (TET) was common, suggesting a possible co-transfer of the corresponding resistance genes. The strain with the broadest resistance profile belonged to ST224, which was shared by three hedgehogs (no. 177, 187, and 113) and, in addition to resistance to SXT, TET, ciprofloxacin (CIP), gentamicin (GN), and AMC, also showed resistance to phenicols, carrying a combination of *floR*, *cmlA,* and *catA* genes. Recent studies indicate a role for *IS6* family transposases in the co-transfer of resistance to these AMs (27). Unfortunately, long-read sequencing, which would allow reconstruction of MGEs potentially associated with this phenotype, was not performed. Although 14 isolates were resistant to CIP, the plasmid-associated gene *qnrB2* was detected in only one of them. Fluoroquinolone (FQ) resistance was observed in isolates from several animals, irrespective of whether they had been treated with MAR. Plasmid replicons IncF, IncI1, and IncX4 were detected in isolates with the broadest resistance profiles, suggesting their involvement in AMR dissemination among strains colonizing different animals at the station.

Findings from a study on wild birds in a rescue station in Italy (28) suggest that resistance to AMC and other beta-lactams is influenced by hospitalization itself, regardless of AM treatment; however, this pattern did not apply to other antimicrobials, such as MAR. In a subsequent Italian study, the same authors (9) reported an increase in the carriage of resistant *E. coli* in rescued hedgehogs over time, indicating that prolonged stays in such facilities may be a risk factor for acquiring AMR.

Overall, studies on antimicrobial resistance in bacteria colonizing hedgehogs, whether free-ranging or captive, remain extremely scarce, and, to our knowledge, no prior work has integrated genomic data in this context. Several studies have reported the presence of antimicrobial-resistant *Staphylococcus* spp., including *S. aureus*, in hedgehogs, with a high prevalence of *mecC*-positive MRSA lineages, indicating that this species represents an important wildlife reservoir of clinically relevant Gram-positive bacteria within a One Health framework (29). Consistent with this, a genomic analysis of hospitalised hedgehogs admitted to a wildlife health centre documented frequent carriage of *S. aureus*, including *mecC*-positive MRSA lineages, suggesting that clinically managed hedgehogs may represent a relevant, though not necessarily causal, interface for the maintenance of host-adapted staphylococci (30). Fredriksson-Ahomaa et al. (2024) (31) performed whole-genome sequencing of potential foodborne pathogens isolated from wildlife hedgehogs (including *Salmonella* spp., *Campylobacter jejuni*, *Yersinia pseudotuberculosis*, *Yersinia enterocolitica*, *Listeria monocytogenes*, and Shiga toxin-producing *E. coli*), suggesting that these animals can act as reservoirs, but did not focus on antimicrobial resistance. Garcias et al. (13) detected genes encoding ESBL/AmpC beta-lactamases in 34.6% of *E. coli* isolates from wild hedgehogs, with *bla*_CTX-M-15_ being the most prevalent type, and reported a single isolate producing the carbapenemase OXA-48. These findings indicate that resistance levels may vary geographically and suggest that hedgehogs in highly populated areas could acquire MDR strains from the environment. Similarly, high percentages of MDR *E. coli* have been reported in hedgehogs from Iran (32), supporting the view that wildlife may act as reservoirs of resistant bacteria.

Half of all *E. coli* isolates sequenced in our study belonged to phylogenetic groups F (*n* = 11) and D (*n* = 2). This was unexpected, as phylogroups A and B1 are generally considered the most common intestinal commensals in animals (33), while group F is less frequently reported. However, information on the composition of the common intestinal microbiota of hedgehogs remains limited, making it difficult to determine whether this pattern reflects species-specific traits or the effects of captivity and treatment.

Seven sampled animals carried closely related ST457 isolates, recently described as an emerging pathogen in wildlife and food-producing animals (34). Other isolates (ST162, ST224, and ST624) were detected in 2–4 animals and may represent lineages from which MDR human or animal pathogens could emerge (35–38). Two distinct ST162 strains were observed, while ST58, another potentially high-risk type, was detected once and harboured the highest number of VAGs. Shared plasmid replicons across different STs, such as IncX4 and IncI1-I(Alpha) in ST224 and ST212, suggest possible inter-clonal exchange. These findings suggest that rescue stations and similar facilities could facilitate genetic exchange and the potential emergence of high-risk clones, particularly under conditions of antimicrobial exposure and prolonged animal stays.

Across the isolates, 2–9 VAGs were detected, with a higher median in phylogroup F compared to phylogroup B1. No typical pathogenic genotype was observed, but all isolates except two (group D) carried a gene encoding long polar fimbriae (*lpfA*), which is associated with bacterial adhesion and colonization. The virulence gene profile was relatively similar in most isolates and included, above all other colonization and adherence factors, such as *eilA* and *air* genes (associated with enteroaggregative adherence), and in ST58, *iha* (IrgA homologue adhesin) and *astA* (heat-stable enterotoxin). Besides, VAGs typically associated with extraintestinal virulence, such as *iss* and *iroN*, were carried by most isolates irrespective of phylogeny, suggesting the opportunistic pathogenic potential of these strains. While ST624, ST224, and ST162 represent lineages commonly isolated from both wildlife and livestock, especially poultry, they were also reported to cause fatal infections in other hosts (35). On the other hand, ST58 and ST457 are considered widespread emergent extraintestinal pathogens (34, 36). Dominance of an AM-susceptible ST457 strain among our isolates suggests the observed spread is due to specific fitness and colonizing factors rather than the antibiotic selective pressure.

Our genomic analysis revealed multiple sequence types carrying virulence-associated genes and plasmid replicons, including MDR lineages, suggesting a potential for genetic exchange and the possible emergence of high-risk clones. Several of the sequence types and virulence markers identified in this study have previously been reported in isolates from humans, livestock, or companion animals, suggesting that rescue centres may function as transient nodes within a broader network of bacterial exchange rather than as isolated ecological units. These findings underscore the role of hedgehogs as sentinels of environmental resistance and emphasize the importance of continued genomic surveillance in wildlife populations.

The authors recognise certain limitations of this study. The study was based on a relatively small number of animals and *E. coli* isolates and was restricted to a single wildlife rescue centre, which constrains the generalisability of the findings. Each hedgehog was sampled only once, preventing assessment of temporal colonisation dynamics and transmission pathways, and the absence of environmental sampling precludes firm conclusions regarding within-facility transmission. Despite these limitations, the study provided novel genomic insights into antimicrobial-resistant *E. coli* in rehabilitated hedgehogs within a One Health context.

## 5 Conclusion

Our data show that hedgehogs kept in a rescue station in 2020 shared several *E. coli* clones from phylogenetic groups F and B1, mostly MDR and belonging to ST457, ST624, ST224, ST212, ST162 and ST58. A limitation of our study is that each animal was sampled only once, preventing the monitoring of colonization dynamics over time and the identification of the direction of strain transmission. Animals may have arrived already colonized with these strains, or they may have acquired them during their stay. The relatively small sample size further limits generalization. Nevertheless, our findings may reflect processes that likely occur in larger rescue and healthcare facilities: animals arriving colonized with multiple *E. coli* strains, the selection and spread of resistant strains between hospitalized animals, and the release of colonized animals that may then act as sources of AMR bacteria for other wildlife. Although wildlife rescue stations are generally small and often overlooked, they represent an understudied environment with limited standardized biosecurity measures and may serve as hotspots for AMR selection and dissemination. These facilities would benefit from targeted biosafety guidelines adapted to their specific needs.

## Data Availability

The datasets presented in this study can be found in online repositories. The names of the repository/repositories and accession number(s) can be found in the article/Supplementary material.
