# Supplementary material for: Hidden carriers: multidrug-resistant bacteria in hedgehogs from a wildlife rescue centre
Source: Front Vet Sci. 2026 Jan 22;13:1754930. doi: 10.3389/fvets.2026.1754930 (PMC12872530; doi:10.3389/fvets.2026.1754930)
Supplement: Supplementary file 1 [file Table_1.docx]

**Supplementary Table S1:** Antimicrobial resistance phenotype of 69 *E. coli* isolates from faeces of 23 hedgehogs (*Erinaceus europaeus, E. roumanicus*) admitted to a Czech wildlife rescue centre.

| **Hedgehog ID** | **Isolate ID** | **No. of resistances** | **Resistance phenotype** |
| --- | --- | --- | --- |
| **43** | 43a | 8 | Amp, Cf, Cip, Na, S, Su, Sxt, T |
|  | 43b | 8 | Amp, Cf, Cip, Na, S, Su, Sxt, T |
|  | 43c | 8 | Amp, Cf, Cip, Na, S, Su, Sxt, T |
| **61** | 61a | 2 | Amp, Cf |
|  | 61b | 2 | Amp, Cf |
|  | 61c | 2 | Amp, Cf |
| **70** | 70a | 1 | Amp |
|  | 70b | 1 | Amp |
|  | 70c | 7 | Amp, Cf, Cip, Na, S, Su, Sxt |
| **73** | 73a | 7 | Amp, Cf, Cip, Na, S, Su, Sxt |
|  | 73b | 8 | Amp, Cf, Cip, Na, S, Su, Sxt, T |
|  | 73c | 6 | Amp, Cip, Na, S, Su, Sxt |
| **85** | 85a | 2 | Amp, Cf |
|  | 85b | 2 | Amp, Cf |
|  | 85c | 0 | - |
| **88** | 88a | 2 | Amp, Cf |
|  | 88b | 2 | Amp, Cf |
|  | 88c | 2 | Amp, Cf, Fox |
| **99** | 99a | 2 | Amp, Cf |
|  | 99b | 1 | Amp |
|  | 99c | 0 | - |
| **106** | 106a | 0 | - |
|  | 106b | 0 | - |
|  | 106c | 0 | - |
| **113** | 113a | 8 | Amp, Cf, Cip, Na, S, Su, Sxt, T |
|  | 113b | 6 | Amp, Cip, Na, S, Su, Sxt |
|  | 113c | 8 | Amp, Cip, Na, S, Su, Sxt, T |
| **134** | 134a | 1 | Amp |
|  | 134b | 1 | Amp |
|  | 134c | 2 | Amc, Amp |
| **151** | 151a | 7 | Amp, Cip, Na, S, Su, Sxt, T |
|  | 151b | 7 | Amp, Cip, Na, S, Su, Sxt, T |
|  | 151c | 0 | - |
| **164** | 164a | 7 | Amp, Cip, Na, S, Su, Sxt, T |
|  | 164b | 8 | Amp, Cip, Fox, Na, S, Su, Sxt, T |
|  | 164c | 9 | Amp, Cf, Cip, Fox, Na, S, Su, Sxt, T |
| **168** | 168a | 3 | Amp, Na, T |
|  | 168b | 3 | Amp, Na, T |
|  | 168c | 3 | Amp, Na, T |
| **170** | 170a | 4 | Amp, Cf, Na, T |
|  | 170b | 7 | Amp, Cip, Na, S, Su, Sxt, T |
|  | 170c | 0 | - |
| **177** | 177a | 10 | Amp, Cf, C, Cip, Gn, Na, S, Su, Sxt, T |
|  | 177b | 9 | Amp, C, Cip, Gn, Na, S, Su, Sxt, T |
|  | 177c | 10 | Amp, Cf, C, Cip, Gn, Na, S, Su, Sxt, T |
| **187** | 187a | 10 | Amp, Cf, C, Cip, Gn, Na, S, Su, Sxt, T |
|  | 187b | 7 | Amp, Cip, Na, S, Su, Sxt, T |
|  | 187c | 0 | - |
| **188** | 188a | 8 | Amc, Amp, Cip, Na, S, Su, Sxt, T |
|  | 188b | 8 | Amc, Amp, Cip, Na, S, Su, Sxt, T |
|  | 188c | 1 | Amp |
| **190** | 190a | 1 | Cf |
|  | 190b | 0 | - |
|  | 190c | 0 | - |
| **191** | 191a | 1 | Cf |
|  | 191b | 0 | - |
|  | 191c | 1 | Cf |
| **192** | 192a | 0 | - |
|  | 192b | 0 | - |
|  | 192c | 7 | Amp, Cip, Na, S, Su, Sxt, T |
| **193** | 193a | 8 | Cf, C, Cip, Na, S, Su, Sxt, T |
|  | 193b | 5 | Cip, Na, S, Su, Sxt |
|  | 193c | 6 | C, Cip, Na, S, Su, Sxt |
| **194** | 194a | 8 | Amc, Amp, Cip, Na, S, Su, Sxt, T |
|  | 194b | 8 | Amc, Amp, Cip, Na, S, Su, Sxt, T |
|  | 194c | 8 | Amc, Amp, Cip, Na, S, Su, Sxt, T |
| **195** | 195a | 8 | Amc, Amp, Cip, Na, S, Su, Sxt, T |
|  | 195b | 8 | Amc, Amp, Cip, Na, S, Su, Sxt, T |
|  | 195c | 8 | Amc, Amp, Cip, Na, S, Su, Sxt, T |

Note – Amc: amoxicillin-clavulanic acid, Amp: ampicillin, C: chloramphenicol, Cf: cephalothin, Cip: ciprofloxacin, Fox: cefoxitin, Gn: gentamicin, Na: nalidixic acid, S: streptomycin, Su: sulfonamide compound, Sxt: sulfamethoxazole-trimethoprim, T: tetracycline.
